# Continuous noninvasive monitoring of barbiturate coma in critically ill children using the Bispectral™ index monitor

**DOI:** 10.1186/cc6138

**Published:** 2007-09-27

**Authors:** Sandra A Prins, Matthijs de Hoog, Joleen H Blok, Dick Tibboel, Gerhard H Visser

**Affiliations:** 1 Department of Pediatric Surgery, Intensive Care Unit, Erasmus MC, University Medical Center, Sophia Children's Hospital, Dr. Molewaterplein 60, 3015 GJ, Rotterdam, The Netherlands; 2 Department of Pediatrics, Intensive Care Unit, Erasmus MC, University Medical Center, Sophia Children's Hospital, Dr. Molewaterplein 60, 3015 GJ, Rotterdam, The Netherlands; 3 Department of Clinical Neurophysiology, Erasmus MC, University Medical Center, 's Gravendijkwal 230, 3015 CE, Rotterdam, the Netherlands

## Abstract

**Introduction:**

Traumatic brain injury and generalized convulsive status epilepticus (GCSE) are conditions that require aggressive management. Barbiturates are used to lower intracranial pressure or to stop epileptiform activity, with the aim being to improve neurological outcome. Dosing of barbiturates is usually guided by the extent of induced burst-suppression pattern on the electroencephalogram (EEG). Dosing beyond the point of burst suppression may increase the risk for complications without offering further therapeutic benefit. For this reason, careful monitoring of EEG parameters is mandatory. A prospective study was conducted to evaluate the usefulness of the bispectral index suppression ratio for monitoring barbiturate coma.

**Methods:**

A prospective observational pilot study was performed at a paediatric (surgical) intensive care unit, including all children with barbiturate-induced coma after traumatic brain injury or GCSE. The BIS™ (Bispectral™ index) monitor expresses a suppression ratio, which represents the percentage of epochs per minute in which the EEG was suppressed. Suppression ratios from the BIS monitor were compared with suppression ratios of full-channel EEG as assessed by quantitative visual analysis.

**Results:**

Five patients with GCSE and three patients after traumatic brain injury (median age 11.6 years, range 4 months to 15 years) were included. In four patients the correlation between the suppression ratios of the BIS and EEG could be determined; the average correlation was 0.68. In two patients, suppression ratios were either high or low, with no intermediate values. This precluded determination of correlation values, as did the isoelectric EEG in a further two patients. In the latter patients, the mean ± standard error BIS suppression ratio was 95 ± 1.6.

**Conclusion:**

Correlations between suppression ratios of the BIS and EEG were found to be only moderate. In particular, asymmetrical EEGs and EEGs with short bursts (less than 1 second) may result in aberrant BIS suppression ratios. The BIS monitor potentially aids monitoring of barbiturate-induced coma because it provides continuous data on EEG suppression between full EEG registrations, but it should be used with caution.

## Introduction

Traumatic brain injury (TBI) and generalized convulsive status epilepticus (GCSE) are conditions that require aggressive management. Barbiturates are used to stop epileptiform activity, with the aim being to improve neurological outcome. Other effects of high barbiturate levels are reduced cerebral metabolism and blood flow, which also are favourable in the treatment of severe epilepsy [1]. Barbiturate therapy also has serious adverse effects, however, in particular cardiovascular depression and hypotension [2,3]. Dosing of barbiturates is guided by the extent of induced burst-suppression pattern on the electroencephalogram (EEG) [4]. Dosing beyond the point of burst suppression may increase the risk for complications without offering further therapeutic benefit [3]. For this reason, careful monitoring of EEG parameters is mandatory.

Several methods of monitoring barbiturate coma are available: interval or continuous EEG monitoring, and regular testing of barbiturate blood levels. In 10 adult patients, Winer and coworkers [5] demonstrated that continuous EEG monitoring was the best modality because it showed the presence of burst suppression on a moment-to-moment basis. They also found poor correlations between serum and cerebrospinal fluid barbiturate levels at any given time, suggesting that barbiturate levels are difficult to interpret because of inter-individual differences in distribution and metabolism [5]. Another possibility is that barbiturate levels are difficult to interpret because of changes in receptor sensitivity [6].

When EEG is used to determine the optimal depth of a barbiturate coma, the goal is to induce a burst suppression pattern [5]. A practical drawback of the standard EEG recording method is that recording and interpretation requires qualified EEG technicians and a clinical neurophysiologist. In addition, most centres do not have the facilities to monitor EEGs and have the EEG interpreted by qualified clinical neurophysiologists continuously for hours to days or even weeks [7-9].

In summary, clinical evaluation of a pentobarbital coma is difficult; barbiturate blood levels may not be reliable and continuous full-channel EEG monitoring is not feasible in many centres, as in ours. Hence, monitoring of a barbiturate coma using the Bispectral™ index (BIS™; Aspect Medical Systems, Newton, MA, USA) monitor is an interesting possibility. This monitor provides a suppression ratio (SR-BIS) and raw EEG traces, which are continuously displayed, thus enabling monitoring of cerebral function. The BIS monitor is relatively easy to use, and nurses and physicians can be taught how to interpret recordings.

We hypothesized that if the optimal SR-BIS values and EEG trace displayed on the BIS monitor are similar to the full channel EEG and remain stable, then the BIS monitor could be used to monitor the SR continuously. If supplemented by a full-channel EEG once a day, this device could replace the need for continuous full-channel recordings. Against the background of the scarcity of data on barbiturate-induced coma in children [10], we opted to conduct a study to explore the usefulness of the BIS monitor during barbiturate-induced coma in critically ill children who require intensive neuro-monitoring. For this purpose, BIS recordings were compared with standard full-channel EEG recordings.

## Materials and methods

### Patients

We conducted a prospective observational pilot study at the paediatric surgical intensive care unit (ICU) and the paediatric ICU of our level-three children's hospital. Because of the strictly observational and noninvasive nature of the study, the institutional review board waived the need for approval. Annually, our paediatric surgical PSICU admits some 10 patients with a Glasgow Coma Score of 8 or less after TBI, which is considered an indication for intracranial pressure monitoring. In about half of these patients, it is necessary to induce a barbiturate coma, after all other methods to decrease intracranial pressure have failed [11]. In addition, every year our paediatric ICU admits three to four patients with refractory GCSE for treatment of their condition with barbiturate coma. All children with TBI or GCSE in whom a barbiturate coma was induced from November 2002 until July 2004 were eligible for inclusion in this study. Patients with TBI facing imminent brain death were not included.

### Procedure

After admission to the ICU, the child's neurological status was evaluated using a standard 24-channel EEG. Barbiturate comas were induced on clinical grounds, independent of the present study. Subsequently, EEGs as well as barbiturate blood levels were requested and repeated on the basis of clinical signs or changes in medication. There is no validated therapeutic range for barbiturate plasma levels; the levels were monitored mainly to avoid toxic concentrations. After informed parental consent, BIS electrodes were applied as described below during the course of the barbiturate coma. All other interventions were recorded.

### Bispectral™ index monitor

We used an A-2000 BIS™ index monitor (version 3.12; Aspect Medical Systems), with commercially available BIS™ paediatric sensor strips with three electrodes. One electrode is placed on the centre of the forehead, one directly above and parallel to the eyebrow, and one in the temple area. The BIS monitor is regularly used in anaesthesiology to quantify the hypnotic effects of anaesthetic drugs by means of a processed cortical two-channel EEG. The monitor uses Fourier transformation and bispectral analysis to compute a number (BIS value) ranging from 0 (isoelectric) to 100 (fully awake). In addition, the EEG recorded by the BIS is continuously displayed (BIS-EEG), together with the device's estimate of the SR. The SR calculated by the BIS (SR-BIS) represents the percentage of epochs during the preceding 63 seconds in which the EEG signal is considered to be suppressed.

The algorithm within the BIS monitor sets limits for electrode impedance and signal quality, and no BIS and SR-BIS values are displayed if the signal has too many artifacts. The standard settings of the device were used for artifact rejection. For offline analysis, all BIS data were downloaded to a laptop computer using the WINHIST and WINLOG program provided by the manufacturer of the BIS monitor.

### Electroencephalogram

The EEG was recorded using silver-silver chloride electrodes attached to the skin with Elefix at electrode positions defined by the International 10–20 system (16 channels; Fp1/2, F7/8, T3/4, T5/6, O1/2, F3/4, C3/4 and P3/4). The EEG was digitally recorded (sample frequency 512 Hz, -3 dB bandpass filter settings 0.13 to 70 Hz) using a BrainlaB device (OSG, Rumst, Belgium). The EEG was visually assessed and for each 10 second EEG epoch, total duration of suppression of cerebral activity (amplitudes below 20 μV) was measured. Subsequently, the SR was calculated as percentage of EEG suppression during 1 minute (SR-EEG), as closely matched to the corresponding BIS epoch as possible (see below). Of EEG registrations lasting more than 1 hour, the first 11 minutes of every full hour were captured, and the SR-EEG was calculated from these data.

### Data management

Relevant clinical data during the treatment period were recorded. Drugs administered during the pentobarbital coma were abstracted from an electronically guided patient data management system.

**Figure 1:**
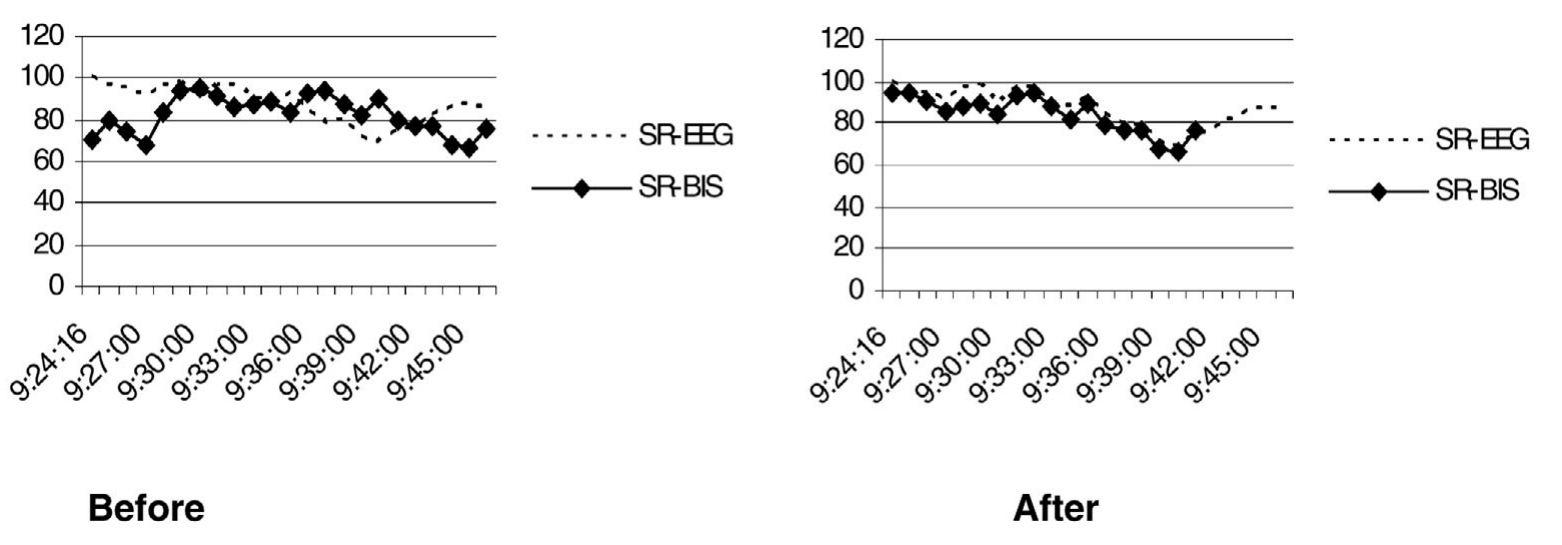
Effect of synchronization. For patient 6, correlation between Bispectral™ index suppression ratio (SR-BIS) and electroencephalographic suppression ratio (SR-EEG) in one EEG was poor (-0.003). After they were synchronized, moving the SR-BIS values 5 minutes back in time, the correlation improved to 0.92.

Synchronization between the SR-BIS and SR-EEG data proved to be a challenge. There appeared to be differences in the algorithms used to determine SR-BIS and SR-EEG. The algorithm of the BIS monitor seems to be less accurate in detecting burst offset than a visual assessor, which led to an underestimation of the SR-BIS. Synchronization was established in several ways. First, we synchronized the computer clocks of the BIS monitor and EEG equipment so that recordings could be linked. Second, in the first four included patients, the software available at that time did not allow recording and exportation of the raw EEG data. Therefore, in these patients we matched patterns in SR-BIS and SR-EEG so that their correlation in time was optimal (Figure 1). For this purpose, we compared SR-BIS with the six SR-EEG datasets that could be determined from the above-mentioned 10 second epochs. That is, the first set was calculated over full minutes running from 0:00 to 1:00, 1:00 to 2:00 and so on until 9:00 to 10:00, yielding 10 SR-EEG values (or more if only a single EEG file of less than 1 hour's duration was available). The second set consisted of SR-EEG values obtained from epochs running from 0:10 to 1:10, 1:10 to 2:10 and so on until 9:10 to 10:10, whereas the last set was based on epochs running from 0:50 to 1:50, 1:50 to 2:50 and so on until 9:50 to 10:50. With this approach, the maximal dysynchrony between SR-BIS and SR-EEG is 5 seconds. In the last four patients, the BIS monitor's raw EEG was captured using a laptop with WINLOG software (provided by Aspect Medical Systems).

### Statistical analysis

The data were analyzed using SPSS for Windows (version 10.0; SPSS Inc., Chicago, IL, USA). The correlation between the SR-BIS and SR-EEG during burst suppression was tested using the Spearman rho correlation coefficient. In case of bimodal data, the correlation was calculated over subsets of data [12]. These subsets of data were found in two patients whose EEGs showed either continuous epileptic activity (SR-EEG <40) or (some) suppression (SR-EEG ≥40), whereas no registrations with intermediate SR-EEG values were available. Statistical differences were considered significant if *P *< 0.05. Correlations from 0.80 to 1.00 were considered large [13].

## Results

Eight patients were included over a period of 18 months. Three patients received barbiturates after TBI and five received barbiturates to treat GCSE. Patient characteristics are listed in Table 1. Raw BIS EEG data were collected from patients 1, 3, 5 and 6 (the last four included patients).

**Table 1:** Patient characteristics

Patient	Age	Sex	Diagnosis	Outcome	Medication other than pentobarbital	Duration of barbiturate coma	Maximum barbiturate blood level
1	4 months	Male	GCSE after asphyxia	P	Midazolam, valproic acid	9 days	20 mg/l (day 2)
2	3 years	Male	GCSE due to Lennox-Gastaut syndrome	M	Lamotrigine, topiramate, valproic acid	3 days	37 mg/l (day 3)
3	3.5 years	Female	GCSE due to viral encephalitis	D	Midazolam, carbamazepine, phenytoin, topiramate	14 days	70 mg/l (day 12)
4	11 years	Male	TBI (hit by baseball bat)	D	Propofol	5 days	-
5	12 years	Female	GCSE next to mental retardation	D	Midazolam	2 days	193 mg/l (day 7)
6	12 years	Male	GCSE due to viral encephalitis	D	Valproic acid, midazolam	>3 weeks	83 mg/l (day 6)
7	15 years	Male	TBI (hit by car)	F	Midazolam, morphine, propofol, fentanyl	16 hours	54 mg/l (day 2)
8	15 years	Male	TBI (hit by car)	M	Morphine	23 hours	47 mg/l (day 2)

D, death; F, full recovery; GCSE, generalized convulsive status epilepticus; M, minor neurological impairment; P, major neurological impairment; TBI, traumatic brain injury.

### Correlation between SR-BIS and SR-EEG

**Figure 2:**
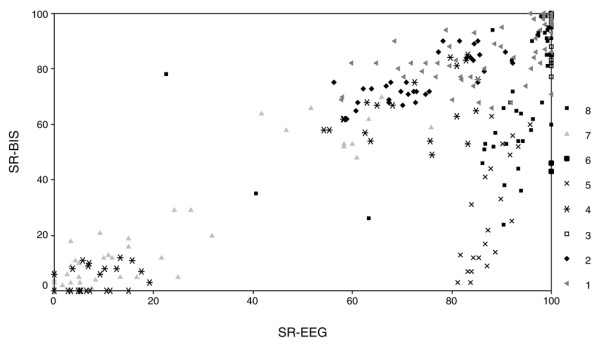
Scatter plot of SR-BIS versus SR-EEG for all eight patients. BIS, Bispectral™ index; EEG, electroencephalogram; SR, suppression ratio.

**Figure 3:**
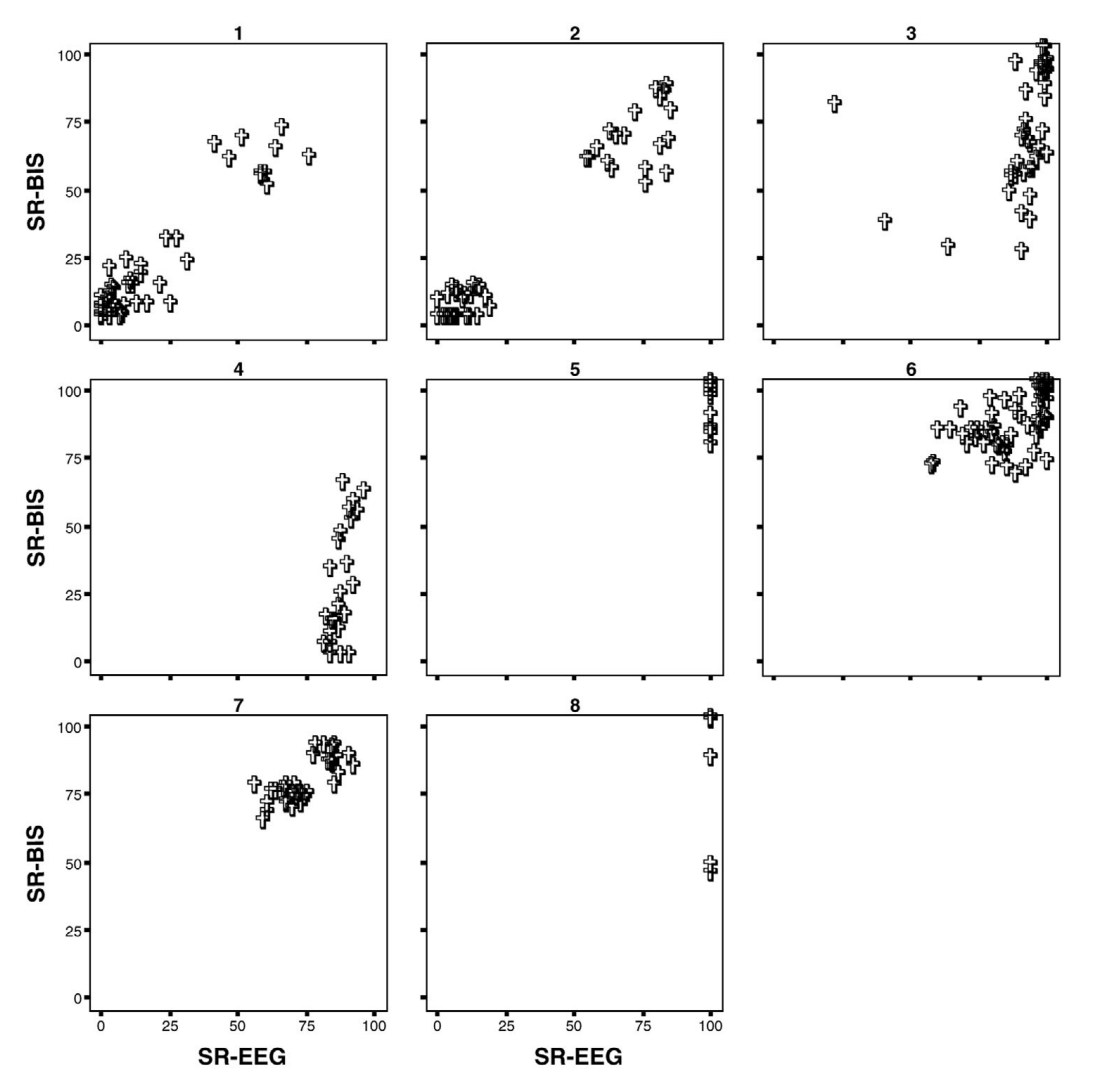
Scatter plots SR-BIS versus SR-EEG for individual patients during burst suppression. BIS, Bispectral™ index; EEG, electroencephalogram; SR, suppression ratio.

The paired observations of all patients are shown in Figure 2. Correlations between SR-BIS and SR-EEG could be calculated for four patients only (patients 3, 4, 6 and 7). The individual correlations between SR-BIS and SR-EEG for these patients were 0.67, 0.64, 0.70 and 0.70, respectively. In patients 1 and 2 the SR distribution was bimodal, as shown by the two 'data clouds' (Figure 3). This precluded determination of reliable correlation values, as did the isoelectric EEG (SR-EEG = 100 and constant) in patients 5 and 8. In the latter patients, SR-BIS ranged from 43 to 100 (mean ± standard deviation 95 ± 1.6).

For patients 1 and 2, correlations between SR-BIS and SR-EEG were calculated for the relevant subsets of data (individual clouds in Figure 3). The highest correlations in these patients were 0.5 and 0.4, respectively.

**Figure 4:**
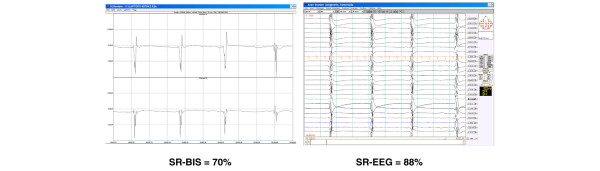
Burst suppression pattern with short-duration bursts in patient 3. The Bispectral™ index suppression ratio (SR-BIS) algorithm yields a value that represents an underestimate of the true electroencephalographic (EEG) suppression.

In a patient with a burst-suppression pattern with bursts of less than 1 second duration (patient 3), SR-BIS tended to underestimate the suppression ratio (Figure 4).

### SR-EEG and barbiturate blood levels

A total of 11 barbiturate blood levels in eight patients with corresponding SR-EEG values were available. The barbiturate blood levels ranged from 18 to 33 mg/l (mean 24 mg/l) and corresponding SR-EEG values ranged from 55 to 100. In the two patients with an isoelectric EEG, blood levels ranged from 15 to 33 mg/l.

## Discussion

The aim of this study was to evaluate the usefulness of the BIS monitor during a barbiturate coma in paediatric ICU patients, as proposed by Arbour [9] and Jaggi and coworkers [14]. We found its application as a continuous monitor of the burst suppression pattern to be promising. The BIS monitor is relatively easy to use, and nurses and physicians can easily be taught how to interpret recordings. SR-BIS and recorded EEG traces are continuously displayed, thus enabling continuous monitoring of cerebral function.

The continuously displayed real-time raw EEG traces correlated well with the full-channel EEG, both at bedside and at comparison between the EEG of the BIS and the full-channel EEG afterward. However, correlations between SR-BIS and SR-EEG were found to be only moderate. To some extent this might have resulted from suboptimal synchronization, but it is likely that some of the discrepancy is caused by differences in the algorithms used to determine SR-BIS and SR-EEG. For example, the algorithm employed by the BIS monitor appears to overestimate the length of the burst and therefore underestimates the SR-BIS (Figure 4 [patient 3]). This underestimation might be caused by the EEG signal's slow return to baseline after a high-amplitude burst. That is, the computerized BIS algorithm may be less accurate in detecting burst offset than a visual assessor. The effects of this bias are more pronounced in situations with many bursts of short duration (less than 1 second) than in a situation with equal SR-EEG but only a few long-duration bursts. However, visually the BIS traces corresponded well with real-time EEG in all patients.

Additional caution should be in taken in cases in which the EEG is (or might be expected to become) asymmetrical. Because the BIS monitor is applied to only one side of the head, significant changes may be overlooked or correlation between SR-BIS and SR-EEG may be poorer than expected. This was illustrated in a patient who had suffered a TBI, resulting in intracranial haemorrhage on the left side of the head (patient 4). His EEG was asymmetrical and, because SR-BIS was recorded over the right side, the correlation between SR-BIS and SR-EEG was low (0.64). In these and similar cases, the best option appears to be simultaneous application of two BIS monitors or to use a 'baseline' EEG to indicate where is the optimal placement for the BIS electrodes.

Barbiturate blood levels within the normal range corresponded with SR-EEG values ranging from 55 to 100 (that is, a brain that is electrically silent at least half of the time). Children with an isoelectric EEG (SR-EEG = 100) had barbiturate blood levels ranging from 15 to 33 mg/l. Apart from showing these children's individual susceptibility to barbiturates, these findings support the assertion by Winer and coworkers [5] that blood levels are inappropriate for titrating barbiturates.

Our study has several limitations. First, although we managed to include most eligible patients presenting to our unit, the group size is small because of the rare requirement for barbiturate-induced coma. In this respect, it should be noted that our hospital serves as a level three paediatric ICU and regional trauma centre (1,100 admissions a year; reference area 4 × 10^6 ^inhabitants), implying that not many units will admit more patients who require a barbiturate coma. This in turn suggests that larger studies should be designed as multicentre projects. Second, we did not monitor EEGs continuously because of organizational limitations. This significantly reduced the amount of available data.

## Conclusion

Based on the experience gained from this pilot study, we suggest that the following protocol be used in future applications. First, a patient's brain function must be evaluated using a full-channel EEG, combined with BIS monitoring, on an individual basis. This combination should be employed to dose barbiturates and to familiarize all those who are involved in evaluating the relation between EEG patterns and visual display of the BIS EEG trace in that particular patient. If the optimal dosage has been established, and if the corresponding EEG trace and concomitant BIS trace remain stable, then a full-channel EEG once a day is probably sufficient to check and evaluate dosage and settings. A new EEG must be taken if there are significant changes in the EEG pattern of the BIS or SR-BIS values, or if there are changes in the clinical situation or medication. Under these conditions, the additional advantages of continuous full-channel EEG probably do not outweigh the practical barriers to this modality. Of course, for objective evaluation of the safety and efficacy of barbiturate induced comas in children, larger prospective studies are required, combining pharmacokinetic and pharmacodynamic studies with continuous EEG and BIS monitoring.

## Key messages

• The BIS monitor provides continuous data on EEG suppression and potentially assists in the monitoring of barbiturate-induced coma in children.

• An EEG must be applied if there are significant changes in EEG pattern, BIS, or SR-BIS values, or if there are changes in clinical situation or medication.

• Larger prospective studies are required that combine pharmacokinetics and pharmacodynamics with continuous EEG and BIS monitoring to determine the safety and efficacy of barbiturate-induced comas in children.

## Abbreviations

BIS™ = Bispectral™ index; EEG = electroencephalogram; GCSE = generalized convulsive status epilepticus; ICU = intensive care unit; SR = suppression ratio; TBI = traumatic brain injury.

## Competing interests

The authors declare that they have no competing interests.

## Authors' contributions

SAP carried out the study, analyzed and interpreted the data, and drafted the manuscript. MdH participated in the design of the study and in the interpretation of the data. JHB participated in the interpretation of the data and helped to draft the manuscript. DT conceived the study and participated in its design. GHV participated in the design of the study and in the management of the data.
